# Spectral Domain Optical Coherence Tomography Assessment of Macular and Optic Nerve Alterations in Patients with Glaucoma and Correlation with Visual Field Index

**DOI:** 10.1155/2018/6581846

**Published:** 2018-10-08

**Authors:** Alessio Martucci, Nicola Toschi, Massimo Cesareo, Clarissa Giannini, Giulio Pocobelli, Francesco Garaci, Raffaele Mancino, Carlo Nucci

**Affiliations:** ^1^Ophthalmology Unit, Department of Experimental Medicine, University of Rome Tor Vergata, Rome, Italy; ^2^Department of Biomedicine and Prevention, University of Rome Tor Vergata, Rome, Italy; ^3^Department of Radiology, Athinoula A. Martinos Center for Biomedical Imaging, Boston, MA, USA; ^4^Harvard Medical School, Boston, MA, USA

## Abstract

**Introduction:**

To evaluate the sectorial thickness of single retinal layers and optic nerve using spectral domain optic coherence tomography (SD-OCT) and highlight the parameters with the best diagnostic accuracy in distinguishing between normal and glaucoma subjects at different stages of the disease.

**Material and Methods:**

For this cross-sectional study, 25 glaucomatous (49 eyes) and 18 age-matched healthy subjects (35 eyes) underwent a complete ophthalmologic examination including visual field testing. Sectorial thickness values of each retinal layer and of the optic nerve were measured using SD-OCT Glaucoma Module Premium Edition (GMPE) software. Each parameter was compared between the groups, and the layers and sectors with the best area under the receiver operating characteristic curve (AUC) were identified. Correlation of visual field index with the most relevant structural parameters was also evaluated.

**Results and Discussion:**

All subjects were grouped according to stage as follows: Controls (CTRL); Early Stage Group (EG) (Stage 1 + Stage 2); Advanced Stage Group (AG) (Stage 3 + Stage 4 + Stage 5). mGCL TI, mGCL TO, mIPL TO, mean mGCL, cpRNFLt NS, and cpRNFLt TI showed the best results in terms of AUC according classification proposed by Swets (0.9 < AUC < 1.0). These parameters also showed significantly different values among group when CTRL vs EG, CTRL vs AG, and EG vs AG were compared. SD-OCT examination showed significant sectorial thickness differences in most of the macular layers when glaucomatous patients at different stages of the disease were compared each other and to the controls.

## 1. Introduction

Primary open-angle glaucoma, a leading cause of blindness in the world, is an optic neuropathy characterized by the death of ganglion cells of the retina, which is associated with the loss of axons that make up the optic nerve. These ultrastructural alterations gradually progress becoming clinically evident as an increased excavation of the optic disc and the presence of specific visual field (VF) defects [1]. Diagnosing and monitoring disease progression is therefore essential for the management of patients with glaucoma. Given that a significant structural loss usually precedes detectable function loss [2], technologies and strategies able to quantify glaucomatous changes at an early stage have the potential to impact prognosis and hence influence quality of life [3]. In this context, spectral domain-optical coherence tomography (SD-OCT) provides a tool for macular segmentation and thickness evaluation of individual retinal layers as well as retinal nerve fiber layer thickness (RNFLt) and Bruch's membrane opening (BMO)-minimum rim width (MRW) assessment. The patented Anatomic Position System (APS) creates an anatomic map of each patient's eye using the center of the fovea and the center of BMO as landmarks. In turn, this allows accurate localization and hence highly sensitive assessment of structural changes.

In this study, sectorial thickness values of each retinal layer at macular level, circumpapillary RNFLt of the optic nerve, and BMO-MRW were measured using SD-OCT Glaucoma Module Premium Edition (GMPE) software (Heidelberg Engineering, Germany) to assess the putative thickness differences between controls and initial glaucoma, controls and advanced glaucoma, and initial and advanced glaucoma.

## 2. Methods

In this cross-sectional study, 49 eyes of 25 glaucomatous patients and 35 age-matched healthy eyes of 18 subjects were recruited from the Glaucoma Clinic and the General Outpatients clinic (respectively) at the University Hospital “Policlinico Tor Vergata” (Rome, Italy). Patients and controls were aged 61.86 ± 6.79 and 60.58 ± 9.22 years, respectively. The study protocol was approved by the local institutional review board and adhered to the tenets of the Declaration of Helsinki. All subjects provided written informed consent.

All subjects underwent a complete ophthalmologic examination including the administration of a medical history questionnaire focused on local and systemic treatments and family history of glaucoma, determination of best-corrected visual acuity with logarithmic Early Treatment Diabetic Retinopathy Study visual acuity charts (Precision Vision, la Salle USA), slit-lamp examination of the anterior segment, intraocular pressure (IOP) evaluation using Goldmann applanation tonometry, pachymetry using an ultrasound pachymeter (Pachette DGH500; DGH Technology, Inc., Philadelphia, PA), gonioscopy, and 24-2 Swedish Interactive Threshold Algorithm (SITA) standard visual field (VF) testing. After pupillary dilation, slit-lamp fundus examination and SD-OCT were performed.

All participants met the following inclusion criteria: best-corrected visual acuity >0.1 logMAR, refractive error < ±5 spherical diopters or < ±3 cylindrical diopters, transparent ocular media, and open anterior chamber (Shaffer classification >20°).

The exclusion criteria comprehended previous or active optic neuropathies, retinal vascular diseases, preproliferative or proliferative diabetic retinopathy, macular degeneration, hereditary retinal dystrophy, use of medication that could affect VF, and previous or active neurological, cerebrovascular, or neurodegenerative diseases. Normal tension glaucoma (NTG) patients were also excluded.

A glaucoma diagnosis was defined, following the European Glaucoma Society criteria [4], as the presence of an elevated IOP (>21 mmHg), marked excavation of the optic nerve head with thinning of the neural rim, notching, focal or diffuse atrophy of neural rim, cup/disc ratio (CDr) in the vertical meridian >0.6, CDr asymmetry between the eyes >0.2, optic disc haemorrhages, denuded circumlinear vessels, and the presence of typical VF defects.

### 2.1. Visual Field Examination

VF examination was performed using Humphrey Swedish Interactive Threshold Algorithm (SITA) standard visual fields with 24-2 test point pattern (Carl Zeiss Meditec Inc., Dublin, CA). As reported in the literature [5], Standard Automated Perimetry (SAP) examinations were considered unreliable and discarded if fixation losses were >20%, false-positive errors >15%, and false-negative errors were >33%. The minimal glaucomatous abnormality was defined as the presence of pattern deviation probability plots with <5%, more than three of which contiguous and one of which <1%, corrected pattern standard deviation or pattern standard deviation significant at *p* < 0.05, or glaucoma hemifield test outside normal limits [5]. VFs were confirmed in at least 3 subsequent VF examinations. For this study, VFs were classified according to the glaucoma staging system based on the visual field index (VFI) [5]. VFI was found to be in excellent correlation with MD across the spectrum of glaucomatous visual loss [5]. The VFI expresses the amount of visual field loss as a percentage relative to the sensitivity of a reference group of healthy observers. To reduce the potentially confounding effects of cataract, the VFI disregards reductions in sensitivity unless they are associated with a pattern deviation probability outside normal limits. Locations at which the pattern deviations are within the 95th percentile of healthy observers are treated as normal and assigned a value of 100%. In addition, locations in the center of the visual field are more heavily weighted and therefore make a greater contribution to the VFI than do those in the periphery. This classification has been deemed easy to use, accurate, and its staging performance has been reported to be either equal or superior to other existing glaucoma staging systems [5].

All subjects were subsequently grouped according to stage as follows: Controls (CTRL); Initial Stages Group (IG) (Stage 1 + Stage 2); Advanced Stages Group (AG) (Stage 3 + Stage 4 + Stage 5).

### 2.2. Optical Coherence Tomography Examination

After pupil dilation, all subjects underwent SD-OCT examination with GMPE software (Heidelberg Retinal Engineering, Dossenheim, Germany).

During the initial Anatomic Positioning System (APS) scan, the scanner performs automatic detection of landmarks and automatic alignment of scans relative to the patient's individual fovea-to-BMO center axis, hence improving accuracy and reproducibility measurements and overcoming measurement errors due to head tilt and eye rotation. Moreover, custom TruTrack™ technology actively tracks the eye during imaging with simultaneous dual-beam imaging minimizing motion artifacts. The GMPE, unlike the previous software versions, offers multi-layer segmentation for assessment of the isolated retinal layers providing a thorough assessment of the macular region via single layer thickness maps, APS, and BMO-MRW-based optic nerve head (ONH) evaluation.

**Figure 1:**
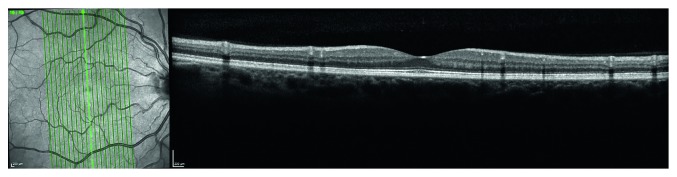
Sample Spectralis OCT posterior pole vertical-oriented scan lines (PPoleV scan) protocols.

**Figure 2:**
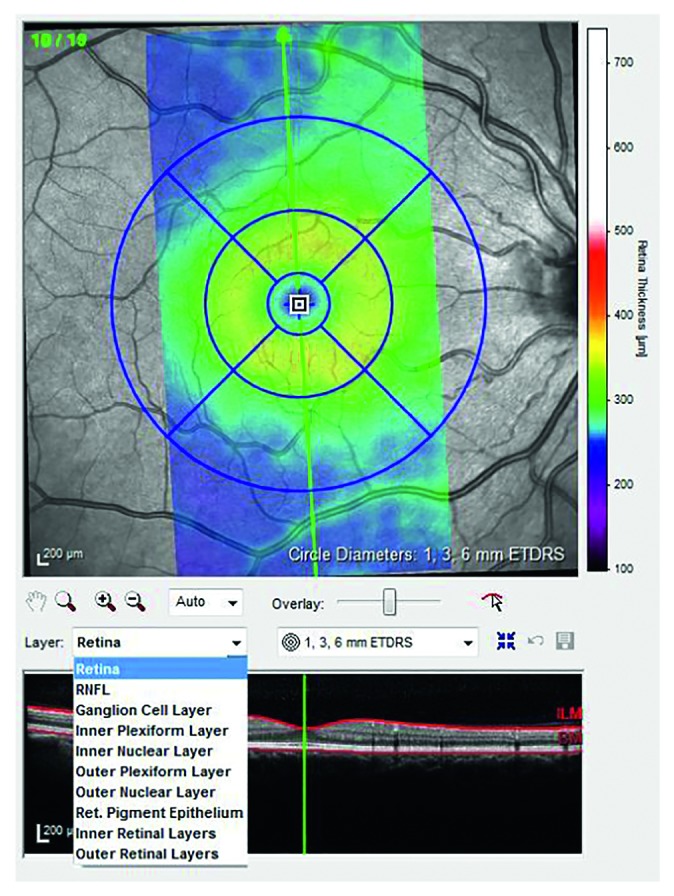
Sample of segmentation of retinal layers.

To obtain perifoveal volumetric retinal scans, both eyes of all subjects were examined using the Spectralis OCT posterior pole vertical-oriented scan lines (PPoleV scan) protocols. PPoleV scan includes 19 single vertical axial scans (30° × 15° OCT volume scan), aligned to the individual fovea-to-BMO center axis with 240 microns distance between sections (Figure 1). Segmentation of the retinal layers in each vertical foveal scan was performed automatically using GMPE software for Spectralis OCT. For each layer provided by the new segmentation software (macular total retina (RETINA), Retinal Nerve Fiber Layer (RNFL), Ganglion Cell Layer (GCL), Inner Plexiform Layer (IPL), Inner Nuclear Layer (INL), Outer Plexiform Layer (OPL), Outer Nuclear Layer (ONL), Retinal Pigmented Epithelium (RPE), Inner Retinal Layers (IRL), and Outer Retinal Layers (ORL)), thickness measurements of all sectors, as defined by the Early Treatment Diabetic Retinopathy Study scheme (temporal inner, superior inner, nasal inner, inferior inner, temporal outer, superior outer, nasal outer, and inferior outer), were considered (Figure 2).

**Figure 3:**
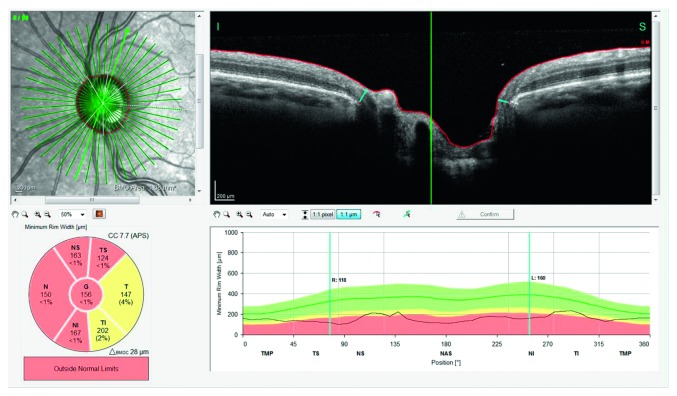
Bruch's membrane opening minimum rim width analysis.

To perform optic nerve head (ONH) analysis, using BMO as the anatomical border of the rim, within 24 scan lines, the GMPE software automatically detects 48 BMO positions along the ONH determining the BMO-based disc margin. BMO-MRW is calculated from the BMO to the nearest point on the internal limiting membrane (ILM). In this study, for each scan, the following BMO-MRW measurements were considered: global, temporal superior, nasal superior, nasal, nasal inferior, temporal inferior, and temporal (Figure 3).

**Figure 4:**
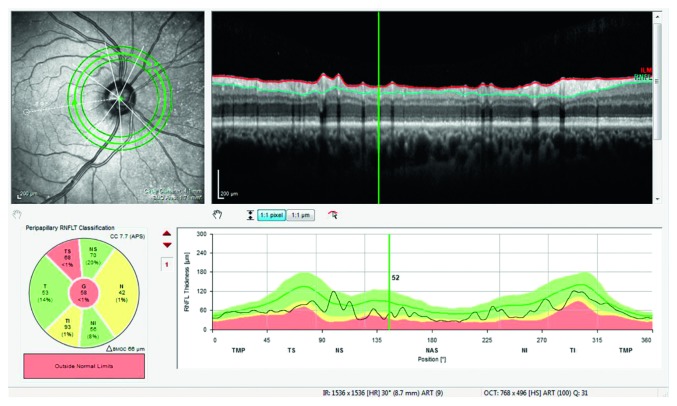
Circumpapillary retinal nerve fiber layer thickness analysis.

Circumpapillary RNFLt (cpRNFLt) analysis is performed acquiring three circle scans automatically centred on the individual fovea-to-BMO center axis ensuring the accurate definition of each single sector independent of head position. For each scan, the central circle has been analyzed and the following cpRNFLt measurements were considered: global, temporal superior, nasal superior, nasal, nasal inferior, temporal inferior, and temporal (Figure 4).

No manual corrections were necessary and only good quality OCT scans, with an OCT score >25, were included in the study.

### 2.3. Statistical Analysis

All data were initially entered into an EXCEL database (Microsoft, Redmond, Washington, United States). Statistical analysis was performed in Statistical Package for the Social Sciences (SPSS) version 23.

Descriptive statistics consisted of the mean ± SD for parameter with Gaussian distributions (after confirmation using the Kolmogorov–Smirnov test), median and interquartile range for variables with nonGaussian distributions.

Comparisons among groups were performed using ANOVA/ANCOVA, for continuous Gaussianly distributed variables, and Mann–Whitney *U* test, for non-Gaussianly distributed variables. *p* < 0.05 was considered statistically significant.

Age comparisons among groups were performed using Friedman ANOVA. A chi-square test was used to test independence among categorical variables.

For all the parameters which showed a statistically significant difference comparing controls vs. initial stages glaucoma, controls vs. advanced stages glaucoma, and initial stages glaucoma vs. advanced stages glaucoma, the diagnostic accuracy was evaluated by fitting a binary logistic regression model and examining the area under the generated receiver operating characteristics (ROC) curve (AUC).

For the interpretation of the AUC values, we referred to the classification proposed by Swets and only retained parameters with high diagnostic accuracy [6]:

- AUC = 0.5, the test is not informative
- 0.5 < AUC < 0.7, the test is not accurate
- 0.7 < AUC < 0.9, the test is moderately accurate
- 0.9 < AUC < 1.0, the test is highly accurate

The correlation between functional, VFI, and structural loss, layers, and sector thicknesses was determined using Spearman's *r* correlation coefficient. Spearman's *r* correlation coefficient was classified accordingly [7]:

- 0.9 to 1.0 (−0.9 to −1.0): very high positive (negative) correlation
- 0.7 to 0.9 (−0.7 to −0.9): high positive (negative) correlation
- 0.5 to 0.7 (−0.5 to −0.7): moderate positive (negative) correlation
- 0.3 to 0.5 (−0.3 to −0.5): low positive (negative) correlation
- 0.0 to 0.3 (−0.0 to −0.3): negligible correlation

## 3. Results

A total of 84 eyes were included in the study. Control group was constituted of 35 eyes (age 60.83 ± 1.53), while initial and advanced stages glaucoma groups were constituted of 22 eyes (age 63.23 ± 1.34) and 27 eyes (age 60.52 ± 1.37), respectively.

There were no statistically significant differences in terms of age (*p*=0.58) and gender (*p*=0.087) among groups.

At macular level, the mean thicknesses of GCL superior, inferior, temporal, and nasal in the inner (GCL mean inner) and outer (GCL mean outer) sectors showed a highly accurate diagnostic ability in all the comparisons considered (CTRL vs. IG, AUC = 0.9; CTRL vs. AG, AUC = 1.0; IG vs. AG, AUC = 0.9). The same result was obtained when macular GCL temporal inner and outer and IPL temporal inner thicknesses were evaluated (CTRL vs. IG, AUC = 0.9; CTRL vs. AG, AUC = 1.0; IG vs. AG, AUC = 0.9). The mean of macular RNFL superior, inferior, temporal, and nasal outer sectors thickness (RNFL mean outer) also showed a highly accurate diagnostic ability in discriminating among all the groups considered for the study (CTRL vs. IG, AUC = 0.9; CTRL vs. AG, AUC = 1.0; IG vs. AG, AUC = 0.9). Moreover, at macular level, both the mean GCL and mean RNFL thickness values, given by the respective mean of superior, inferior, temporal, and nasal inner and outer sectors, resulted highly accurate in discriminating among all the groups (CTRL vs. IG, AUC = 0.9; CTRL vs. AG, AUC = 1.0; IG vs. AG, AUC = 0.9) (Table 1).

At ONH level, circumpapillary RNFL global and circumpapillary RNFL temporal superior sector thicknesses resulted highly accurate in discriminating among all the groups considered for the study (CTRL vs. IG, AUC = 0.9; CTRL vs. AG, AUC = 1.0; IG vs. AG, AUC = 0.9) (Table 1).

Table 2 reports descriptive statistics of the parameters that showed the highest diagnostic ability in all the following comparison: CTRL vs. IG, CTRL vs. AG, and IG vs. AG.

The correlation between functional, VFI, and structural loss thickness of the OCT parameters has been determined using Spearman's *r* correlation coefficient.  Table 3 reports the OCT parameters that showed high and very high correlation coefficient with VFI (see Supplementary Material for the other parameters). Interestingly, all the parameters that showed the highest AUC also showed a positive correlation with VFI. In particular, if we consider the parameters that showed the best AUC values, at macular level, GCL mean inner (*r* = 0.81), GCL mean outer (*r* = 0.75), GCL temporal inner (*r* = 0.79), GCL temporal outer (*r* = 0.72), IPL temporal inner (*r* = 0.78), RNFL mean outer (*r* = 0.71), mean GCL (*r* = 0.76), and mean RNFL (*r* = 0.73) were highly positively correlated with VFI. On the contrary, circumpapillary RNFLt global (*r* = 0.45) and circumpapillary RNFLt temporal superior (*r* = 0.52) showed a low positive correlation with VFI.

## 4. Discussion

OCT technology has been proposed more than 20 years ago for noninvasive cross-sectional imaging in biological systems [8], and the first generation of time domain OCT has been superseded by the newest spectral domain instruments. Given that SD-OCT provides an accurate reconstruction of ONH and of the macular area, hence highlighting the presence of possible structural damage, it is increasingly becoming part of common clinical practice. Moreover, thanks to the faster scanning speed and the increased axial resolution, the latest OCT generation offers high resolution images which are less affected by eye movement artifacts and are more reproducible [9–11]. In this regard, local intrasession and intersession variability in OCT have been previously described as very low and uniform across eyes and layers [12, 13].

Before Zeimer et al. [14] suggested the use of macular imaging for glaucoma evaluation, the most important clinical parameter was the circumpapillary RNFL thickness [15–20]. However, due to the presence of blood vessels and the high variability of the ONH structure (even among healthy subjects), circumpapillary RNFLt measurements may not always be completely reliable in glaucoma diagnosis [21]. Instead, the macula is a relatively simple structure, constituted by multiple layers whose structure is not influenced by the presence of blood vessels. The shape of the macula, and more specifically the RGC layer, is generally less variable among healthy individuals as compared to other ocular structures such as the RNFL and ONH. Thus, in conjunction with the fact that it contains 50% of the total retinal ganglion cells (RGCs) and 35% of RNFL, macula appears to be a promising area for glaucoma evaluation [14, 21].

While previous studies with TD-OCT reported a good diagnostic ability of macular thickness, sensitivity and specificity were lower than those provided by circumpapillary RNFLt [22–28].

Using the fast-macular cube scan of a prototype software, Martinez-de-la-Martinez-de-la-Casa et al. [29] observed that with the macular segmentation software provided the Spectralis OCT, macular RNFLt measurements performed better than other algorithms in discriminating healthy subjects from glaucoma suspects. A similar result was also found considering RNFL + GCL + IPL thickness and total macular thickness [30]. Another study by Mathers et al. [31] also highlighted the usefulness of macular thickness as measured by SD-OCT in confirming the existence and extent of VF defects.

In a previous study using 3D-OCT 2000 (Topcon Corp., Tokyo, Japan), a thickness reduction of macular layers with progression of glaucoma was reported. This study showed a significantly lower ganglion cell-inner plexiform layer thickness (GCIPLT), as well as lower macular ganglion cell complex thickness (GCCT: a combination of macular RNFL thickness and GCIPLT) in POAG patients, compared to normal controls, progressing with the severity of glaucomatous damage [32].

In this study, we evaluated the ability of the parameters assessed using the GMPE software at macular and optic nerve level to discriminate among the following groups: controls versus initial glaucoma, controls versus advanced glaucoma, and initial versus advanced glaucoma. GMPE is a specific software for glaucoma diagnosis that, unlike other software, allows a layer by layer and sector by sector macular assessment. Thanks to this software, we are able to obtain, at macular level, macular total retina, retinal nerve fiber layer, ganglion cell layer, inner plexiform layer, inner nuclear layer, outer plexiform layer, outer nuclear layer, retinal pigmented epithelium, inner retinal layers, and outer retinal layer thickness measurements in all sectors as defined by the Early Treatment Diabetic Retinopathy Study scheme (temporal inner, superior inner, nasal inner, inferior inner, temporal outer, superior outer, nasal outer, and inferior outer). In addition, for the purpose of the study, we calculated and evaluated the mean thickness value for each layer.

ONH was also assessed using the GMPE software and retinal nerve fiber layer thickness, and Bruch's membrane opening minimum rim width was obtained.

Of all the parameters, the presence of statistically significant differences among the groups was evaluated. Subsequently the diagnostic ability of those parameters to discriminate controls versus initial glaucoma, controls versus advanced glaucoma, and initial versus advanced glaucoma was tested using AUC. Subsequently, only the parameters with good ability to discriminate among groups in all of the three comparisons were considered. At macular level, the parameters that best performed were mean GCL, GCL mean inner and outer, GCL temporal inner and outer, IPL temporal inner, mean RNFL, and RNFL mean outer.

At ONH level, the parameters that best performed were circumpapillary RNFL global and temporal superior. None of the Bruch's membrane opening parameters had very high diagnostic accuracy in discriminating among all the groups.

Interestingly, the median of all the parameters discussed above showed reducing thickness values trend with progression of the disease.

Although many efforts have been made in the development of new technologies to diagnose and evaluate the glaucoma progression, clinicians still base their decisions on standard “white-on-white” automated VF testing, which remains the best-studied way to assess disease progression. Nevertheless, many confounding factors, such as media opacity, may affect the results. Most importantly, distraction, or other factors involving the patient's participation make VF tests unreliable. This leads to a high level of disagreement among clinicians whether or not glaucoma is progressing in their patients.

The VFI is a new global metric that represents the entire VF as a single percentage of normal. Based on an aggregate percentage of visual function with 100% being a perfect age-adjusted visual field, it assigns a number between 1% and 100%. Central VF points are more heavily weighted, and the percentage of VF loss is calculated based on pattern or total deviations depending on the depth of loss. Interestingly, the progression rates calculated by VFI are much less affected by cataract development and cataract surgery than the traditional mean deviation index or the pattern standard deviation [33, 34].

Due to the clinical relevance of VFI, its possible correlation with the OCT parameters has been assessed in this study. Interestingly, most of the OCT parameters showed a correlation with VFI. In particular, macular parameters such as GCL mean inner, GCL temporal inner, GCL superior inner, IPL temporal inner, IPL mean inner, GCL inferior inner, mean GCL, GCL mean outer, RNFL nasal outer, mean RNFL, GCL temporal outer, GCL nasal outer, GCL nasal inner, RNFL mean outer, and IPL inferior inner showed from high to very high correlation with VFI. Thus, suggesting their possible usefulness in the objective evaluation of the progression of the disease.

Interestingly, our data go beyond those of a previous study that showed a good diagnostic ability of macular GCL temporal inner thicknesses in discriminating controls vs. initial glaucoma [35]. In fact, our results suggest that this parameter had, not only a good ability in discriminating controls from initial glaucoma, but also controls from advanced glaucoma and initial glaucoma from advanced glaucoma. Moreover, this parameter showed a very high positive correlation with VFI, showing a strong structure-function correlation, thus further supporting a possible usefulness of OCT parameters in glaucoma diagnosis and follow-up.

There is much interest in the world of scientific research regarding the use of OCT in the diagnosis and follow-up of glaucoma, which is now considered a real neurodegenerative disease [36, 37]. Many studies have focused on the analysis of the diagnostic capacity of this tool in discriminating healthy subjects from subjects with suspected glaucoma or at the initial stage of the disease. Our work has been aimed at pushing beyond searching parameters that would allow, not only an early diagnosis of the disease but also a patient evaluation during lifetime, allowing to discriminate among the patients at the initial stage of the disease and those at an advanced stage.

There are several limitations to this prospective study. The cohort included a small number of patients, and this may have affected our analysis. Moreover, while we aimed to include patients with a broad range of glaucoma severity, the group of patients affected by advanced stages of disease was smaller than all other groups, possibly affecting statistical power.

In conclusion, this study suggests a possible usefulness of macular segmentation and ONH analysis with GMPE in the evaluation of glaucoma patients. Our data suggest that OCT may be a useful tool in detecting macular microstructural changes related to the progression of glaucoma and that this tracking is possible since the early stages of the disease. Our initial results warrant further prospective longitudinal studies on a larger cohort to confirm the ability of OCT parameters, to track disease progression in glaucoma, and eventually test new neuroprotective agents in the management of glaucoma [38, 39].

## Figures and Tables

**Table 1:** Mann–Whitney and AUC of the parameters with the highest diagnostic ability in the CTRL vs. IG, CTRL vs. AG, and IG vs. AG comparisons.

CTRL vs. IG			CTRL vs. AG			IG vs. AG		
Parameter	AUC	*p* value	Parameter	AUC	*p* value	Parameter	AUC	*p* value
Macula GCL mean inner	0.9	4.29449*E* − 06	Macula GCL mean inner	1.0	3.94592*E* − 10	Macula GCL mean inner	0.9	9.29422*E* − 06
Macula GCL mean outer	0.9	2.3797*E* − 06	Macula GCL mean outer	1.0	3.11259*E* − 10	Macula GCL mean outer	0.9	4.87372*E* − 07
Macula GCL temporal inner	0.9	6.40501*E* − 07	Macula GCL temporal inner	1.0	3.59673*E* − 10	Macula GCL temporal inner	0.9	3.5386*E* − 06
Macula GCL temporal outer	0.9	2.26545*E*− 07	Macula GCL temporal outer	1.0	1.6719*E* − 10	Macula GCL temporal outer	0.9	4.12819*E* − 06
Macula IPL temporal inner	0.9	4.81767*E* − 06	Macula IPL temporal inner	1.0	1.67747*E* − 10	Macula IPL temporal inner	0.9	1.42929*E* − 05
Macula RNFL mean outer	0.9	1.6614*E* − 05	Macula RNFL mean outer	1.0	4.46656*E* − 10	Macula RNFL mean outer	0.9	3.41429*E* − 06
Macula mean GCL	0.9	9.86195*E* − 07	Macula mean GCL	1.0	1.78465*E* − 10	Macula mean GCL	0.9	1.04181*E* − 06
Macula mean RNFL	0.9	2.51857*E* − 06	Macula mean RNFL	1.0	3.14624*E* − 10	Macula mean RNFL	0.9	9.886*E* − 07
cpRNFL global	0.9	3.19772*E* − 06	cpRNFL global	1.0	2.68425*E* − 10	cpRNFL global	0.9	7.41664*E* − 07
cpRNFL temporal superior	0.9	2.83685*E* − 06	cpRNFL temporal superior	1.0	2.70908*E* − 10	cpRNFL temporal superior	0.9	4.33798*E* − 06
VFI	0.9	2.14775*E* − 06	VFI	1.0	3.81295*E* − 11	VFI	1.0	6.5955*E* − 09

GCL: ganglion cell layer; IPL: inner plexiform layer; cpRNFL: circumpapillary retinal nerve fiber layer thickness; VFI: visual field index; CTRL: control group; IG: Early Glaucoma Group; AG: Advanced Glaucoma Group; *p* value Mann–Whitney <0.05.

**Table 2:** Descriptive statistics of the parameters with the highest diagnostic ability in the CTRL vs. IG, CTRL vs. AG, and IG vs. AG comparisons.

	CTRL			IG			AG		
Parameter	Median	Interquartile range low	Interquartile range high	Median	Interquartile range low	Interquartile range high	Median	Interquartile range low	Interquartile range high
Macula GCL mean inner	52.00	50.25	54.75	43.13	39.25	45.75	27.75	22.50	36.50
Macula GCL mean outer	35.50	32.75	38.50	30.13	27.75	32.25	22.50	20.75	25.75
Macula GCL temporal inner	51.00	47.00	53.00	35.00	29.00	42.00	20.00	18.00	27.00
Macula GCL temporal outer	39.00	35.00	40.00	26.50	24.00	31.00	19.00	17.00	22.00
Macula IPL temporal inner	43.00	40.00	45.00	34.00	32.00	37.00	25.00	22.00	27.00
Macula RNFL mean outer	34.75	33.50	37.00	28.88	25.25	31.50	21.00	19.00	24.50
Macula mean GCL	31.59	29.27	33.20	27.34	26.06	27.88	21.41	19.80	24.14
Macula mean RNFL	40.94	38.61	42.73	32.03	26.06	35.78	20.66	19.52	25.16
cpRNFL global	90.00	79.00	97.00	61.50	58.00	72.00	47.00	39.00	51.00
cpRNFL temporal superior	113.00	101.00	138.00	79.50	66.00	96.00	39.00	31.00	57.00
VFI	99.00	98.00	99.00	92.00	86.00	96.00	49.00	13.00	66.00

GCL: ganglion cell layer; IPL: inner plexiform layer; cpRNFL: circumpapillary retinal nerve fiber layer thickness; VFI: visual field index; CTRL: control group; IG: Early Glaucoma Group; AG: Advanced Glaucoma Group.

**Table 3:** Pearson correlation coefficient between VFI and OCT parameters.

Parameter	Correlation with VFI (*r*)
Macula GCL mean inner	0.81
Macula GCL temporal inner	0.79
Macula GCL superior inner	0.78
Macula IPL temporal inner	0.78
Macula IPL mean inner	0.77
Macula GCL inferior inner	0.76
Mean GCL	0.76
Macula GCL mean outer	0.75
Macula RNFL nasal outer	0.75
Mean RNFL	0.73
Macula GCL temporal outer	0.72
Macula GCL nasal outer	0.71
Macula GCL nasal inner	0.71
Macula RNFL mean outer	0.71
Macula IPL inferior inner	0.70

GCL: ganglion cell layer; IPL: inner plexiform layer; VFI: visual field index; CTRL: control group; IG: Early Glaucoma Group; AG: Advanced Glaucoma Group; *r*: Pearson correlation coefficient.

## Data Availability

The data used to support the findings of this study are included within the text and the supplementary information file.

## Supplementary Materials

Supplementary Materials In the supplementary materials, AUC and Mann–Whitney *p* values for each comparison, complete descriptive statistics, and correlation of each parameter with VFI are reported.
